# Coregulatory Networks Remodel the Disease-Specific Functions of Orphan Nuclear Receptor TR4

**DOI:** 10.3390/cells15131218

**Published:** 2026-07-03

**Authors:** Yunlong Liu, Qing Yu, Shuyuan Cheng, Mengtian Ren, Xiuping Fu

**Affiliations:** 1 School of Life Sciences, Tiangong University, Tianjin 300387, China; 2313920214@tiangong.edu.cn (Q.Y.); 2313920223@tiangong.edu.cn (S.C.); mengtianren@tiangong.edu.cn (M.R.); fuxiuping@tiangong.edu.cn (X.F.); 2 Cangzhou Institute of Tiangong University, Cangzhou 061000, China

**Keywords:** TR4, orphan nuclear receptor, coregulatory networks, transcriptional plasticity, precision medicine

## Abstract

Testicular receptor 4 (TR4, NR2C2) is an orphan nuclear receptor involved in the regulation of metabolism, inflammation, cardiovascular disease, and cancer. Accumulating evidence indicates that TR4 exhibits functional plasticity, exerting protective or pathogenic effects depending on tissue and disease context, and sometimes displaying opposing roles within the same disease. However, the mechanisms underlying this functional duality remain poorly understood. Recent studies indicate that TR4 activity is determined not only by the receptor itself but also by dynamic coregulatory networks. Through interactions with coactivators, corepressors, epigenetic regulators, and environmental signaling pathways, TR4 integrates metabolic cues to generate context-dependent transcriptional programs. Coactivator networks centered on PGC-1α, steroid receptor coactivator (SRC) family members, and CBP/p300 support oxidative metabolism and anti-inflammatory responses, whereas RIP140-, NCoR/SMRT-, and HDAC-associated networks promote lipid accumulation, chronic inflammation, fibrosis, and tumor progression. Regulators such as JAZF1 further influence TR4 activity by reshaping coregulator recruitment and target-gene selection. In this review, we summarize the structural basis of TR4 regulation and discuss how coregulatory network remodeling governs its functions in metabolic, cardiovascular, inflammatory, and malignant diseases. We propose that TR4 functions as a context-dependent transcriptional platform whose activities are defined by its coregulatory landscape, providing a framework for precision therapies.

## 1. Introduction

Nuclear receptors constitute a large family of ligand-regulated transcription factors that play central roles in coordinating cellular responses to environmental and physiological cues. By sensing intracellular and extracellular signals and reprogramming gene expression networks, nuclear receptors govern diverse biological processes, including metabolic homeostasis, inflammatory responses, cell fate determination, and tumorigenesis [1]. In contrast to classical ligand-dependent nuclear receptors, orphan nuclear receptors lack well-defined endogenous ligands and therefore rely more heavily on dynamic interactions with transcriptional cofactors and regulatory networks for functional control [2]. TR4, an important member of the orphan nuclear receptor family, contains a canonical DNA-binding domain (DBD) and ligand-binding domain (LBD), enabling it to regulate a broad range of transcriptional programs through recognition of specific TR4REs [3,4]. Increasing evidence has demonstrated that TR4 participates in lipid metabolism, glucose homeostasis, mitochondrial function, and inflammatory regulation, while also influencing tumor metabolic reprogramming, cell proliferation, epithelial–mesenchymal transition (EMT), cancer stemness, and other pathological processes [5,6,7]. These observations position TR4 as an important transcriptional node linking metabolic dysfunction, chronic inflammation, and tumor development.

Despite its broad biological functions, the role of TR4 in human disease is characterized by remarkable complexity and functional duality [8,9]. In metabolic disorders, TR4 has been reported to maintain metabolic homeostasis by promoting fatty acid oxidation and improving insulin sensitivity, yet under different conditions it may enhance lipid accumulation and chronic inflammation, thereby accelerating the progression of obesity and non-alcoholic fatty liver disease (NAFLD) [10]. This paradox is even more evident in cancer. For example, in prostate cancer, TR4 has been shown to facilitate androgen receptor (AR)-associated signaling, castration resistance, and metastatic progression, while under specific genetic or cellular contexts, it may exert tumor-suppressive functions [8]. Notably, opposite biological effects have been observed even within the same disease setting, depending on metabolic status, inflammatory milieu, or epigenetic background [11,12,13]. Such context-dependent observations have generated seemingly contradictory conclusions across studies and highlight the limitations of the traditional “one gene–one function” paradigm in explaining TR4 biology.

Accumulating evidence suggests that the functional output of TR4 is not intrinsically fixed but is dynamically shaped by the coregulatory networks in which it operates [14,15]. As a transcription factor, TR4 depends on coordinated interactions with coactivators, corepressors, and chromatin-remodeling complexes to execute gene regulatory programs [16,17,18]. Importantly, these coregulatory networks influence not only the magnitude of TR4 transcriptional activity but also its target-gene selectivity, chromatin occupancy, and downstream transcriptional outputs. For instance, coactivator networks centered on peroxisome proliferator-activated receptor γ coactivator-1α (PGC-1α), SRC family members, including SRC-1 (NCOA1), SRC-2 (NCOA2), and SRC-3 (NCOA3), and CREB-binding protein /E1A binding protein p300 (CBP/p300) preferentially promote transcriptional programs associated with oxidative metabolism, mitochondrial function, and anti-inflammatory responses [19,20]. In contrast, corepressor networks involving receptor-interacting protein 140 (RIP140/NRIP1), nuclear receptor corepressor (NCoR) and silencing mediator of retinoid and thyroid hormone receptors (SMRT), and histone deacetylases (HDACs) favor transcriptional states linked to lipid accumulation, inflammatory activation, and tumor progression [21,22]. Furthermore, environmentally responsive regulators, including juxtaposed with another zinc finger protein 1 (JAZF1), non-coding RNAs (ncRNAs), and inflammatory signaling mediators, provide additional layers of tissue-specific and disease-specific control over TR4 activity [23,24]. Collectively, these findings suggest that TR4 should be viewed as a transcriptional regulatory platform whose functional consequences are determined by its surrounding coregulatory landscape rather than by the receptor alone.

This emerging concept reflects a broader paradigm shift in nuclear receptor biology. Increasingly, disease-associated transcriptional outputs appear to be governed not only by receptor abundance or activation status but also by the regulatory context in which receptors function. Consequently, understanding TR4 biology requires moving beyond the descriptive question of “which diseases involve TR4” toward a mechanistic framework centered on the “TR4–coregulatory network–transcriptional program” axis. Such a perspective provides a unified explanation for the apparently contradictory roles of TR4 across metabolic diseases, cancer, and chronic inflammatory disorders, while also revealing common transcriptional principles shared among these pathological conditions. More importantly, it suggests that the functional duality of TR4 may primarily arise from dynamic alterations in the coregulator landscape across tissues and disease states rather than from inherent changes in TR4 itself. In other words, the central question in TR4 biology is gradually shifting from “what TR4 is” to “under which coregulatory network TR4 functions.”

In this review, we systematically examine TR4 from the perspective of its coregulatory networks. We first summarize the structural organization of TR4, its DNA-recognition mechanisms, and the molecular basis underlying its context-dependent transcriptional regulation. We then discuss the major coactivator, corepressor, and environmentally responsive regulatory networks associated with TR4, with particular emphasis on how distinct combinations of coregulators reshape TR4 functions in metabolic disorders, cancer, and inflammation-associated diseases. In addition, we highlight the contribution of epigenetic mechanisms to TR4 transcriptional plasticity and discuss their potential therapeutic implications. Finally, we propose a conceptual framework in which TR4 functions not as a receptor with intrinsically fixed biological properties but as a dynamic transcriptional regulatory platform whose disease-specific activities are continuously defined by changing coregulatory networks. This perspective not only provides a unifying explanation for the functional duality of TR4 but also offers a theoretical foundation for precision therapeutic strategies targeting nuclear receptor-associated diseases.

Literature included in this review was identified primarily through PubMed, Web of Science, and Scopus using combinations of the keywords “TR4”, “NR2C2”, “orphan nuclear receptor”, “coregulator”, “coactivator”, “corepressor”, “PGC-1α”, “RIP140”, “HDAC”, “JAZF1”, “metabolism”, “cancer”, “inflammation”, and “epigenetics”. Relevant English-language articles were selected based on their relevance to the molecular mechanisms, coregulatory networks, and disease-specific functions of TR4.

## 2. Structural Basis for Context-Dependent Transcriptional Regulation of TR4

TR4 belongs to the NR2C subfamily of nuclear receptors and is classified as a canonical orphan nuclear receptor. Similar to other members of the nuclear receptor superfamily, TR4 regulates gene expression through recognition of specific DNA response elements and recruitment of transcriptional regulatory complexes [25]. However, unlike classical ligand-dependent nuclear receptors, no universally accepted endogenous ligand has yet been identified for TR4. Consequently, its transcriptional activity appears to be governed predominantly by the composition of intracellular coregulatory networks and the local chromatin environment rather than by ligand-induced activation [26]. Understanding the structural organization and transcriptional regulatory mechanisms of TR4 is therefore essential not only for elucidating its biological functions but also for explaining its remarkable functional plasticity across different physiological and pathological contexts.

### 2.1. Structural Architecture of TR4 and Its Transcriptional Regulatory Framework

**Figure 1:**
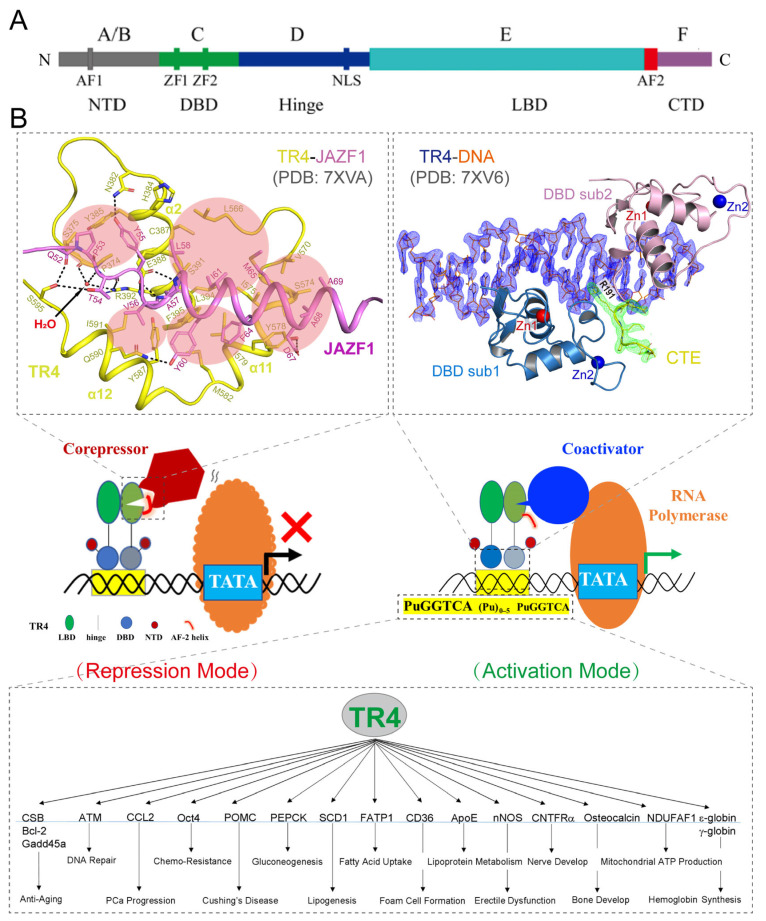
Structural Basis and Coregulator-Dependent Transcriptional Regulation of TR4. (**A**) Domain organization of TR4, including the N-terminal activation function-1 (AF-1), DBD, hinge region containing the nuclear localization signal (NLS), LBD, and C-terminal activation function-2 (AF-2). (**B**) Crystal structures of the TR4–JAZF1 complex (PDB: 7XVA) and the TR4–DNA complex (PDB: 7XV6) reveal the molecular basis of coregulator recruitment and target DNA recognition [20]. TR4 can function either as a transcriptional activator or repressor depending on the recruited cofactors. Through interactions with distinct coactivator and corepressor complexes, TR4 regulates a broad spectrum of target genes involved in metabolism, inflammation, DNA repair, cellular differentiation, and tumor progression [3]. Adapted from [3,20].

TR4 exhibits the characteristic modular architecture of nuclear receptors, comprising an N-terminal activation domain (A/B region), a DBD, a hinge region, an LBD, and a C-terminal functional region (Figure 1A).

The A/B region contains the ligand-independent activation function-1 (AF-1) domain, which interacts with promoter-associated transcriptional cofactors to facilitate gene activation [22]. Although this region displays relatively low sequence conservation, accumulating evidence suggests that it contributes substantially to tissue-specific transcriptional regulation by TR4 [22].

The DBD represents the most highly conserved region of the receptor and contains two canonical C4 zinc-finger motifs responsible for sequence-specific DNA recognition and binding, thereby forming the molecular basis of TR4-mediated transcriptional regulation (Figure 1B). Located between the DBD and LBD, the hinge region possesses considerable structural flexibility and contains a nuclear localization signal (NLS), which contributes to receptor conformational dynamics and nuclear trafficking [27].

The LBD constitutes another critical regulatory module of TR4. Early crystallographic studies revealed that the ligand-free TR4 LBD adopts an auto-inhibited conformation in which portions of the ligand-binding pocket are occluded by C-terminal helices and the coactivator-binding interface remains partially inaccessible [26]. This structural arrangement may prevent nonspecific receptor activation and suggests that TR4 utilizes a regulatory mechanism distinct from that of classical ligand-activated nuclear receptors. Rather than relying primarily on ligand-induced conformational switching, TR4 appears to depend more heavily on coregulator recruitment and protein-mediated structural remodeling.

Recent advances in structural biology have substantially expanded our understanding of TR4 regulation (Figure 1B) [20]. High-resolution structural analysis of the TR4–JAZF1 complex demonstrated that JAZF1 binds a specific surface pocket within the TR4 LBD and induces local conformational rearrangements that stabilize a unique α13 helix, thereby reducing coactivator recruitment and attenuating TR4 transcriptional activity. Notably, the α13 helix is a distinctive structural feature currently identified only within the TR2/TR4 receptor subfamily, suggesting the existence of specialized coregulatory mechanisms unique to these receptors [20]. These findings provide a structural basis for JAZF1-mediated regulation of TR4 and further support the concept that TR4 activity can be remodeled through coregulator-induced conformational changes.

Collectively, the structural organization of TR4 enables it not only to recognize target DNA sequences but also to function as an integrative platform for protein–protein interactions. In particular, the pronounced conformational plasticity of the LBD provides an important structural foundation for the context-dependent functional remodeling mediated by distinct coregulatory networks.

### 2.2. DNA Recognition Properties Underlying Broad Transcriptional Competence

The transcriptional activity of nuclear receptors is fundamentally determined by their ability to recognize specific DNA response elements. Structural analyses of the TR4 DBD and TR4–DNA complexes have revealed the molecular basis underlying its unusually broad target-gene recognition spectrum. The recognition helix within the first zinc-finger motif directly engages the DNA major groove, whereas water-mediated hydrogen-bonding networks and local DNA structural features further contribute to binding specificity and stability. Consequently, TR4 employs both conventional base readout and DNA shape recognition mechanisms to achieve selective DNA binding. In addition, dimerization of the TR4 DBD further enhances binding affinity and target-site selectivity (Figure 1B) [20]. Integrating structural and biochemical evidence, TR4 is currently recognized as a highly versatile DNA-binding receptor capable of recognizing direct-repeat response elements composed of two PuGGTCA half-sites across a broad range of DR0–DR5 configurations. Its remarkable tolerance for variable spacer lengths allows for multiple DNA-binding modes and substantially expands its potential genomic target repertoire [28,29].

Compared with many nuclear receptors that exhibit relatively restricted DNA-recognition preferences, TR4 possesses exceptional sequence flexibility and transcriptional versatility, enabling the regulation of genes involved in energy metabolism, inflammatory signaling, cellular proliferation, differentiation, and tissue homeostasis (Figure 1B) [4]. This broad DNA-binding competence provides an important structural foundation for the pleiotropic roles of TR4 across diverse physiological and pathological contexts. Nevertheless, DNA recognition alone cannot account for the striking context-dependent functions of TR4. Accumulating evidence indicates that TR4 may occupy identical genomic loci yet elicit fundamentally distinct transcriptional outcomes in different cellular environments [8]. These observations suggest that the determinants of TR4 functional specificity extend beyond DNA binding itself and are critically governed by the composition and activity of the surrounding coregulatory network, which ultimately shapes target-gene selection and transcriptional output.

### 2.3. Context Dependency as a Defining Feature of TR4 Function

Although TR4 possesses the canonical structural organization and DNA-binding properties of a nuclear receptor, its biological functions are considerably more complex than those of many classical family members. Unlike conventional ligand-activated nuclear receptors, TR4 lacks a clearly established endogenous ligand, and its activity appears to be governed primarily by dynamic protein–protein interaction networks and intracellular signaling states rather than by a single ligand-dependent activation mechanism. As a result, TR4 exhibits remarkable functional plasticity across different physiological and pathological contexts. A central feature of this regulatory flexibility is its dependence on coregulator assembly. TR4 dynamically interacts with diverse coactivators and corepressors, including PGC-1α, SRC family members, CBP/p300, RIP140, NCoR, SMRT, and HDAC-containing complexes, which collectively determine whether TR4 promotes transcriptional programs associated with metabolic homeostasis and anti-inflammatory responses or alternatively drives lipid accumulation, inflammatory signaling, and disease progression [19,20,21,22]. In addition, modulators such as JAZF1 further influence TR4 activity by altering receptor conformation and coregulator recruitment, adding another layer of regulatory complexity [23,24].

Beyond coregulator composition, the transcriptional output of TR4 is profoundly influenced by the surrounding chromatin landscape. Productive TR4-mediated gene regulation depends not only on receptor occupancy at response elements but also on chromatin accessibility, enhancer activity, histone modifications, and the recruitment of chromatin-remodeling complexes [30,31,32]. Consequently, tissue-specific and disease-specific epigenetic environments establish distinct regulatory contexts that shape TR4 target-gene selection and transcriptional outcomes. Collectively, these observations support the view that TR4 functions as a prototypical context-dependent nuclear receptor whose biological effects are determined not by receptor abundance or DNA binding alone, but by the integrated influence of coregulatory networks and epigenetic states. This conceptual framework provides the mechanistic basis for understanding the remarkable functional plasticity—and, in many cases, functional duality—of TR4 across diverse disease settings.

## 3. Coregulatory Networks Governing TR4 Function

### 3.1. Metabolic Coactivator Networks Direct Oxidative Transcriptional Programs

Transcriptional activation by TR4 in metabolic tissues is governed by a hierarchically organized coactivator network. Unlike classical ligand-dependent nuclear receptors whose activity is primarily determined by ligand availability, TR4 is highly responsive to cellular metabolic status, coregulator composition, and chromatin context [14,15,16,17,18]. Although TR4 provides the DNA-binding scaffold for transcriptional complex assembly, the magnitude and direction of transcriptional output are largely dictated by the associated coactivator repertoire. Under oxidative metabolic conditions, a coordinated network centered on PGC-1α/β and reinforced by SRC family members, CBP/p300, and p300/CBP-associated factor (PCAF) promotes transcriptional programs involved in fatty acid oxidation, gluconeogenesis, mitochondrial biogenesis, and energy expenditure (Table 1).

Conceptually, this network can be viewed as a three-tier regulatory hierarchy. TR4 is proposed to function as a metabolic signal integrator that converts upstream nutritional and metabolic cues into altered cofactor recruitment preferences. PGC-1α/β subsequently appears to determine the identity of downstream transcriptional programs, whereas SRC proteins, CBP/p300, and PCAF act as chromatin-remodeling and transcription-amplifying effectors (Table 1). Together, these components are thought to establish an oxidative transcriptional network that enables TR4 to coordinate metabolic adaptation in response to environmental and intracellular cues.

#### 3.1.1. Metabolic Signal Integration Determines TR4 Coactivator Preference

The LBD of TR4 exhibits substantial conformational plasticity and can respond to diverse endogenous metabolic molecules. Polyunsaturated fatty acids, their metabolites, and retinoid-related compounds have been reported to enhance TR4 activity by promoting conformational states favorable for coactivator recruitment [26,33]. Similarly, fatty acids, lipid-derived metabolites, and rosiglitazone stimulate TR4-dependent transactivation of the *CD36* promoter in macrophages [34]. Importantly, these metabolic inputs do not merely switch TR4 activity on or off; rather, they reshape the receptor’s conformational landscape and thereby influence its affinity for specific coregulators. Because the AF-2 region of TR4 does not fully conform to the canonical activation mechanism characteristic of classical ligand-dependent nuclear receptors [26], metabolic signals appear to regulate TR4 primarily by modulating interaction surfaces recognized by coactivators, particularly PGC-1α/β, thereby coupling nutrient availability to selective assembly of transcriptional complexes.

#### 3.1.2. PGC-1 Coactivators Specify Metabolic Transcriptional Outputs

PGC-1α and PGC-1β are central determinants of TR4-dependent transcriptional specificity and function as master regulators of mitochondrial biogenesis, oxidative metabolism, adaptive thermogenesis, and nutrient-responsive gene expression (Table 1) [19,35]. Through direct interaction with TR4, they establish transcriptional specificity while simultaneously serving as molecular platforms for the recruitment of additional coactivators. Evidence from erythroid development demonstrates that PGC-1α/β cooperate with TR2/TR4 at β-globin regulatory regions; whereas deletion of either coactivator alone exerts only modest effects, simultaneous loss of both proteins severely impairs embryonic and adult globin expression, resulting in defective erythropoiesis, anemia, and systemic metabolic abnormalities [36]. In metabolic tissues, PGC-1α further enhances TR4-dependent activation of genes such as *phosphoenolpyruvate carboxykinase* (*PEPCK*) and *apolipoprotein E* (*ApoE*), thereby directing transcriptional outputs toward gluconeogenesis, fatty acid oxidation, and lipoprotein metabolism [36]. Consequently, the presence or absence of PGC-1 coactivators functions as a key determinant of TR4 transcriptional orientation, with PGC-1α/β favoring oxidative and energy-consuming programs, whereas replacement by repressors such as RIP140 redirects TR4 activity toward lipogenic pathways, providing a mechanistic basis for the functional duality of TR4 in metabolic disease.

#### 3.1.3. SRC–CBP/p300–PCAF Complexes Amplify TR4-Dependent Gene Activation

Following establishment of transcriptional specificity by PGC-1 coactivators, the magnitude of TR4-mediated gene activation is amplified by a cooperative module consisting of SRC family members, CBP/p300, and PCAF (Table 1). SRC proteins (SRC-1, SRC-2, and SRC-3) interact with activated TR4 through canonical LXXLL motifs, stabilizing transcriptionally active receptor conformations and enhancing transactivation [20,37,38]. CBP and p300 are subsequently recruited to the complex, where their histone acetyltransferase activities increase chromatin accessibility, facilitate RNA polymerase II recruitment, and further strengthen SRC–TR4 interactions through p300-mediated acetylation of SRC-1 [21,39,40,41]. PCAF provides additional acetyltransferase activity through direct interactions with nuclear receptor DBDs [42,43]. Importantly, assembly of this activation module is dynamically controlled by post-translational modifications. Dephosphorylated TR4 preferentially recruits PCAF and cooperates with SRC proteins and p300 to activate transcription, whereas mitogen-activated protein kinase (MAPK)-mediated phosphorylation of the AF-1 domain promotes recruitment of RIP140 and suppresses metabolic activation programs [22]. This phosphorylation-dependent cofactor switch provides a mechanism by which TR4 dynamically adapts its transcriptional output to changing metabolic conditions.

### 3.2. Corepressor Networks Reprogram TR4 Toward Lipogenic and Pro-Inflammatory States

The conversion of TR4 from a transcriptionally active regulator to a transcriptional repressor is driven by phosphorylation-dependent coregulator switching and reinforced by the assembly of dedicated corepressor complexes. In contrast to the PGC-1–centered coactivator network that promotes oxidative metabolism and energy expenditure, the repressive network redirects TR4 toward lipid storage, chronic inflammation, and disease-associated transcriptional programs. This process follows a hierarchical regulatory cascade in which phosphorylation-induced conformational changes alter TR4 coregulator preference, RIP140 bridges TR4 to HDACs, and NCoR/SMRT–HDAC3 complexes establish stable epigenetic repression (Table 1). Through these coordinated mechanisms, TR4 is reprogrammed from a driver of oxidative metabolism into a regulator of lipogenic and inflammatory transcriptional states.

#### 3.2.1. RIP140 Mediates Phosphorylation-Dependent Transcriptional Switching

RIP140 is a prototypical nuclear receptor corepressor that interacts with LBDs through canonical LXXLL motifs [37]. Recruitment of RIP140 to TR4 is highly dependent on receptor phosphorylation status; MAPK-mediated phosphorylation within the AF-1 domain promotes replacement of the coactivator PCAF by RIP140, thereby shifting TR4 from transcriptional activation to repression (Table 1) [22]. Functional studies have demonstrated that RIP140 suppresses TR4-dependent transactivation of the *Bcl-2* promoter in a dose-dependent manner [44]. Although structural information on the TR4–RIP140 complex remains limited, biochemical evidence showing RIP140 interaction with the closely related orphan receptor TR2 supports a conserved repression mechanism within the NR2C subfamily [45]. Importantly, RIP140 exerts its repressive function through recruitment of HDACs. Its N-terminal repression domain (RD1) directly binds HDAC3, an interaction further enhanced by RIP140 phosphorylation [46]. Similar HDAC-dependent repression mechanisms have been described for several nuclear receptors, including retinoic acid and estrogen receptors (ERs) [47], and structure–function analyses have identified RD1 as the key determinant for HDAC3 recruitment [48]. Physiologically, RIP140 suppresses oxidative metabolic genes such as *Ucp1*, *Cpt1b*, and *Cidea*, whereas RIP140 deficiency derepresses these pathways and enhances fatty acid oxidation [49]. Consistent with these molecular effects, RIP140-null mice display increased energy expenditure, resistance to diet-induced obesity, reduced hepatic steatosis, and a lean phenotype [50]. Collectively, RIP140 functions as a pivotal molecular switch that redirects TR4 activity from oxidative metabolism toward lipid storage and metabolic dysfunction.

#### 3.2.2. NCoR/SMRT–HDAC3 Complexes Establish Epigenetic Silencing Programs

NCoR and SMRT are highly conserved transcriptional repressors that mediate ligand-independent silencing throughout the nuclear receptor superfamily (Table 1). NCoR was initially identified as a core mediator of repression by thyroid hormone and retinoic acid receptors [51], whereas SMRT similarly associates with unliganded receptors to suppress transcription [52]. Mechanistically, SMRT recruits the scaffold protein mSin3A and HDAC1 to establish repressive chromatin structures [53], while subsequent biochemical studies identified HDAC3, TBL1, and GPS2 as integral components of stable SMRT-containing repression complexes [54]. Likewise, NCoR-mediated repression critically depends on HDAC3 catalytic activity [55]. Given the substantial structural homology between the TR4 LBD and those of thyroid hormone and retinoic acid receptors, it is hypothesized that TR4 in a transcriptionally inactive state—or with an unoccupied AF-2 surface—may provide docking interfaces for CoRNR-box-containing corepressors such as NCoR and SMRT. Recruitment of NCoR/SMRT–HDAC3 complexes is thought to consequently direct histone deacetylase activity to TR4 target loci, promoting chromatin compaction and transcriptional silencing. Although direct evidence for TR4-dependent recruitment of these complexes remains incomplete, extensive studies in metabolic tissues suggest the biological plausibility of this mechanism. In the liver, HDAC3 is a central regulator of lipid metabolism, and hepatocyte-specific deletion of HDAC3 causes profound derepression of lipogenic genes and severe hepatic steatosis [56]. These observations suggest that HDAC3 normally maintains metabolic homeostasis through chromatin-based repression. Based on these findings, it is reasonable to propose that excessive recruitment of NCoR/SMRT–HDAC3 complexes to TR4-regulated oxidative loci could suppress fatty acid oxidation and mitochondrial pathways, thereby promoting lipid accumulation and metabolic dysfunction (Table 1). Notably, RIP140-mediated repression and NCoR/SMRT-dependent silencing operate cooperatively rather than independently: RIP140 rapidly displaces coactivators through competitive binding, whereas NCoR/SMRT–HDAC3 complexes establish durable epigenetic repression through chromatin remodeling. Together, these mechanisms drive large-scale transcriptional reprogramming, shifting TR4 from a protective regulator of energy expenditure toward a promoter of lipid accumulation and inflammatory responses, a transition that likely contributes to the pathogenesis of NAFLD, metabolic syndrome, and related metabolic disorders.

### 3.3. Environment-Responsive Regulatory Networks Fine-Tune TR4 Activity

The balance between TR4 coactivator and corepressor networks appears not to be governed by a simple binary switch but is continuously refined by environment-responsive regulators, including JAZF1, ncRNAs, and inflammatory signaling pathways (Table 1). Acting at multiple regulatory levels—from transcriptional complex assembly and post-transcriptional regulation to signaling-dependent protein interactions—these factors constitute an outer regulatory layer surrounding the core TR4 transcriptional machinery. Whereas coactivators and corepressors determine the intrinsic transcriptional state of TR4, environment-responsive regulators are suggested to dynamically recalibrate this state in response to metabolic, inflammatory, and pathological cues, thereby conferring tissue-specific and disease-specific functionality.

#### 3.3.1. JAZF1 Restricts Coregulator Assembly Through Structural Competition

Among known TR4-interacting proteins, JAZF1 is one of the best-characterized context-dependent regulators. Structural analyses of the TR4–JAZF1 complex revealed an unconventional repression mechanism in which the N-terminal TR4-interacting domain (TID) of JAZF1 binds a noncanonical surface within the TR4 LBD formed by helices α3, α12, and the TR2/TR4-specific α13 helix (Figure 1B) [20,23]. Because this interface partially overlaps with the docking site utilized by coactivators such as SRC-1 and CBP, JAZF1 acts as a structural competitor that blocks coactivator recruitment while stabilizing an autoinhibited receptor conformation [20]. Consistent with this mechanism, JAZF1 markedly suppresses TR4-dependent transactivation of DR1-containing reporters with relatively limited effects on other nuclear receptors, highlighting the selectivity of the TR4–JAZF1 axis [23]. Beyond its direct interaction with TR4, JAZF1 also attenuates inflammation and lipid accumulation through inhibition of TAK1 and downstream nuclear factor kappa-light-chain-enhancer of activated B cells (NF-κB) signaling (Table 1) [57]. Collectively, JAZF1 functions as a TR4-selective regulatory node that modulates receptor activity without altering receptor abundance, thereby linking metabolic homeostasis, inflammatory signaling, and nuclear receptor regulation.

#### 3.3.2. NcRNAs Regulate TR4 Abundance at the Post-Transcriptional Level

In contrast to JAZF1-mediated regulation of protein–protein interactions, ncRNAs primarily modulate TR4 function by controlling receptor abundance (Table 1). Through effects on mRNA stability and translational efficiency, microRNAs indirectly influence the capacity of TR4-dependent transcriptional networks to assemble and respond to upstream signals. In renal cell carcinoma (RCC), miR-32-5p directly targets the 3′ untranslated region of TR4 mRNA, suppressing receptor expression, attenuating HGF/MET signaling, and reducing tumor migration and invasion; clinically, decreased miR-32-5p expression is associated with elevated TR4 levels and poor prognosis [24]. Similarly, miR-133a represses TR4 expression in macrophages, thereby limiting oxidized LDL-induced lipid accumulation and foam-cell formation [5]. Given the established role of TR4 in promoting CD36-mediated lipid uptake [34], the miR-133a–TR4 axis may constitute an endogenous protective mechanism against atherosclerotic progression. In prostate cancer, TR4 also transcriptionally suppresses miR-145 to enhance OCT4-mediated stemness and chemoresistance [58]. These findings further illustrate that ncRNAs can act as critical downstream effectors through which TR4 influences both metabolic and oncogenic signaling. Unlike coactivators and corepressors, microRNAs do not directly determine transcriptional directionality; rather, they establish the quantitative boundaries within which TR4-associated regulatory networks operate and thereby modulate overall system responsiveness.

#### 3.3.3. Inflammatory Signaling Rewires TR4 Coregulatory Networks

Inflammatory signaling pathways provide an additional layer of regulation by actively remodeling TR4-associated protein interaction networks and redirecting transcriptional outputs (Table 1). In 3T3-L1 adipocytes, TNF-α suppresses expression of the TR4 target genes *FATP1* and *PC*, resulting in reduced lipid accumulation [59]. Mechanistically, NF-κB physically interacts with TR4 and decreases its affinity for TR4REs, thereby destabilizing receptor occupancy and attenuating TR4-dependent transactivation [59]. Unlike JAZF1, which represses TR4 through structural competition for coactivator-binding surfaces, NF-κB acts primarily by interfering with DNA binding and transcriptional complex stability. Notably, the juvenile hormone analog pyriproxyfen partially restores TR4 target-gene expression and lipid accumulation under TNF-α stimulation, indicating that TR4 activity remains pharmacologically modulatable even in inflammatory settings [59]. Reciprocal regulation between TR4 and NF-κB has also been documented in sepsis, where elevated TR4 expression correlates with poor clinical outcomes, and experimental TR4 overexpression enhances inflammatory cytokine production, whereas TR4 depletion attenuates inflammatory responses [60]. Similarly, in testicular macrophages, TR4 promotes IL-1β and IL-6 expression through activation of NF-κB signaling, establishing a positive feedback circuit that amplifies inflammatory responses [60]. Collectively, these findings indicate that inflammatory signaling does not merely activate or suppress TR4; rather, it rewires the broader TR4 coregulatory landscape, redirecting transcriptional outputs toward pro-inflammatory programs and contributing to the context-dependent functional plasticity that characterizes TR4 biology.

**Table 1:** Major TR4 coregulatory networks and their disease-associated functional outcomes.

Coregulatory Factor/Network	Functional Category	Effect on TR4 Transcriptional Program	Representative Biological Processes	Disease-Associated Outcome
PGC-1α ^a^	Coactivator	Promotes oxidative metabolic gene expression [35,36]	Fatty acid oxidation [35], mitochondrial biogenesis [36], energy homeostasis [61]	Protective effects in metabolic disorders [36]; improved insulin sensitivity [61] and reduced inflammation [36,61]
SRC family (SRC-1/SRC-2/SRC-3) ^a^	Coactivator	Enhances transcriptional activation and chromatin accessibility [37,38,40,62]	Cell proliferation [63,64], EMT, tumor cell survival [64]	Tumor progression and metastasis, particularly in prostate cancer [8,9,64,65]
CBP/p300 ^a^	Coactivator/Histone acetyltransferase	Increases histone acetylation and enhancer activity [39,40,41]	Open chromatin formation, metabolic and anti-inflammatory transcription [40,41]	Supports context-dependent activation of TR4 target genes [20,40]
RIP140 (NRIP1) ^b^	Corepressor	Suppresses oxidative metabolism-related transcription [45,46,49]	Lipid storage, adipogenesis, metabolic repression [49,50]	Obesity, insulin resistance, and NAFLD progression [49,50]
NCoR/SMRT ^b^	Corepressor complex	Recruits transcriptional repression machinery [51,52]	Suppression of metabolic homeostasis genes [55,56]	Chronic inflammation, metabolic dysfunction, and tumor-associated transcriptional states [55,66]
HDACs (especially HDAC3) ^b^	Epigenetic corepressor	Promotes histone deacetylation and chromatin compaction [55,56]	Inflammatory activation, repression of mitochondrial programs [55,56]	NAFLD progression, chronic inflammation, and cancer-promoting transcriptional programs [56,67]
JAZF1 ^a^	Environmental-responsive regulator	Modulates TR4 conformation and coregulator recruitment [20,23,68]	Metabolic adaptation, anti-inflammatory signaling [57,69]	Protective effects in obesity, type 2 diabetes(T2D), and metabolic syndrome [68,69]
Inflammatory signaling pathways (NF-κB, cytokine-associated networks) ^a^	Context-dependent regulatory network	Redirects TR4 target gene selection under inflammatory conditions [59,60]	Immune regulation, inflammatory transcriptional reprogramming [59,60]	Determines pro-inflammatory or anti-inflammatory TR4 functions [60,70]
Chromatin-remodeling complexes ^b^	Epigenetic regulatory network	Reshape TR4 chromatin occupancy and enhancer selection [30,31,71,72]	Cell fate determination, transcriptional plasticity [71,72]	Disease-specific TR4 functional remodeling across metabolic and cancer contexts [8,30]

Evidence levels are defined as follows: ^a^ established experimental evidence; ^b^ evidence inferred from related nuclear receptors.

## 4. Disease-Specific Functions of TR4 Are Defined by Distinct Coregulatory Networks

### 4.1. Metabolic Disorders: Coregulatory Network Switching Determines the Functional Output of TR4

In metabolic tissues, TR4 regulates transcriptional programs governing gluconeogenesis, lipogenesis, and fatty acid uptake through direct binding to TR4REs within promoters of genes such as *PEPCK*, *SCD1*, *FATP1*, and *CD36* [73,74,75]. However, receptor occupancy alone does not determine transcriptional outcomes. Whether TR4 promotes oxidative metabolism or favors lipid storage depends largely on the composition of the associated coregulatory network. Dynamic competition among coactivators, corepressors, and environment-responsive regulators ultimately dictates the direction of TR4-dependent transcriptional programs, providing a mechanistic framework for understanding its functional duality in metabolic diseases.

#### 4.1.1. Insulin Resistance and Type 2 Diabetes

**Figure 2:**
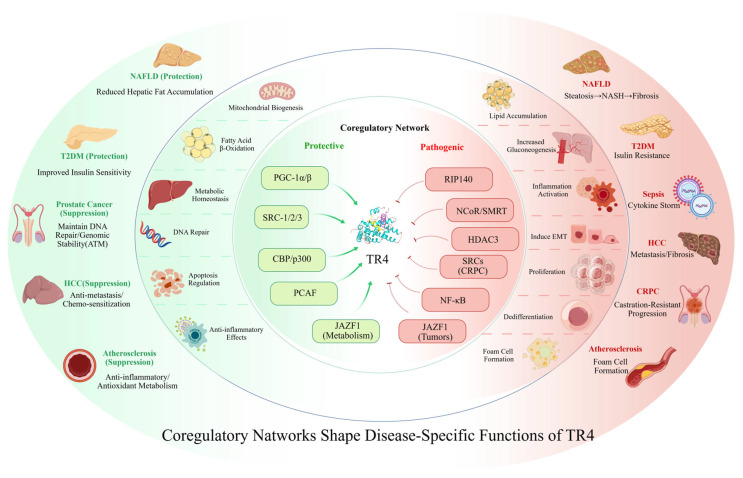
Coregulatory networks shape the disease-specific functions of TR4. TR4 functions as a context-dependent transcriptional regulator whose disease-associated activities are determined by distinct coregulatory networks. Coactivator-dominant networks, including PGC-1α, SRC family members, CBP/p300, and metabolism-associated JAZF1 signaling, promote transcriptional programs involved in fatty acid oxidation, mitochondrial biogenesis, anti-inflammatory responses, and genomic stability, thereby contributing to metabolic homeostasis and tumor-suppressive effects. In contrast, corepressor-dominant networks involving RIP140, NCoR/SMRT, HDAC3, NF-κB signaling, and tumor-associated JAZF1 pathways drive lipogenesis, inflammatory activation, EMT, proliferation, and dedifferentiation, leading to disease progression. The balance between these competing coregulatory networks determines whether TR4 exerts protective or pathogenic functions across metabolic disorders, inflammatory diseases, cardiovascular diseases, and cancer. Created by http://BioGDP.com (accessed on 14 June 2026).

TR4 has been shown to directly control multiple genes involved in hepatic glucose production, fatty acid uptake, and lipid synthesis, including *PEPCK*, *SCD1*, *FATP1*, *CD36*, and pyruvate carboxylase [34,73,74,75,76]. TR4 deficiency impairs gluconeogenesis and lowers fasting glucose levels, whereas TR4 overexpression enhances hepatic glucose output and promotes lipid accumulation [73,74,75,76]. Nevertheless, these metabolic outcomes are determined less by TR4 abundance than by the coregulatory complexes assembled at TR4-bound loci (Figure 2).

(1) The PGC-1α–TR4 axis as a determinant of oxidative metabolic programs

Through its N-terminal LXXLL motif, PGC-1α binds the activated TR4 LBD and recruits SRC family coactivators together with p300/CBP, forming a transcriptional activation complex that promotes histone acetylation, chromatin accessibility, and RNA polymerase II recruitment [35,36,41]. This complex activates genes involved in mitochondrial function, fatty acid oxidation, and metabolic adaptation, including *PEPCK* and *ApoE* [36]. Under physiological conditions, the PGC-1α–TR4 axis supports metabolic homeostasis; however, obesity, insulin resistance, and T2D are frequently associated with reduced PGC-1α expression or activity [35,61]. The PPARGC1A (encoding PGC-1α) Gly482Ser variant further links impaired PGC-1α function to increased T2D susceptibility [77]. Loss of PGC-1α not only diminishes recruitment of SRC/p300 complexes but also exposes the TR4 cofactor-binding surface to competing repressors such as RIP140, thereby redirecting TR4-dependent transcription away from oxidative metabolism and toward lipid accumulation (Figure 2).

(2) The JAZF1–TR4 regulatory axis in metabolic homeostasis

By binding a noncanonical surface within the TR4 LBD through its TR4-interacting domain (TID), JAZF1 competitively blocks recruitment of coactivators including SRC-1 and CBP (Figure 1B) [20,68]. Functionally, JAZF1 overexpression reduces weight gain, hepatic steatosis, and diet-induced hyperglycemia, partly through disruption of PGC-1α/SRC/p300 recruitment to gluconeogenic promoters such as *PEPCK* [57,67]. In parallel, JAZF1 suppresses TAK1-dependent NF-κB signaling and attenuates inflammatory cytokine production [57,60]. The physiological relevance of this pathway is supported by human genetics, as *JAZF1* represents a major T2D susceptibility locus identified by GWAS [69]. Reduced hepatic JAZF1 expression in obesity may therefore weaken repression of TR4-dependent transcription and contribute to excessive hepatic glucose production, linking genetic predisposition to dysregulated TR4 coregulatory networks (Figure 2) [78].

(3) The RIP140/HDAC repressive network and lipid accumulation

RIP140 recruits HDAC3 through its N-terminal repression domain, establishing chromatin-based repression of oxidative metabolic genes via histone deacetylation and chromatin compaction [48,49]. In obesity, NAFLD, and metabolic syndrome, elevated RIP140 expression and HDAC activity favor assembly of this repressive network [49,50]. Although TR4 remains associated with many of the same genomic loci, oxidative genes such as *Ucp1*, *Cpt1b*, and *Cidea* become suppressed, whereas lipogenic pathways driven by *SCD1* and *CD36* remain active [49]. The resulting imbalance between lipid uptake, storage, and oxidation promotes ectopic lipid deposition and insulin resistance (Figure 2) [44,50].

Taken together, the functional role of TR4 in insulin resistance and T2D is dictated primarily by competition among PGC-1α-, JAZF1-, and RIP140-centered regulatory networks rather than by receptor abundance alone. Occupancy of the TR4 cofactor-binding interface acts as the critical molecular determinant: PGC-1α promotes oxidative metabolism, RIP140 favors lipid storage, and JAZF1 restrains excessive metabolic activation. Dynamic remodeling of this network therefore provides the mechanistic basis for the context-dependent functions of TR4 in metabolic disease.

#### 4.1.2. Nonalcoholic Fatty Liver Disease

TR4 exhibits pronounced functional duality in NAFLD, where its biological effects are strongly influenced by the surrounding hepatocellular coregulatory environment. TR4-deficient mice are resistant to age- and diet-induced hepatic steatosis and display reduced expression of genes involved in lipid uptake, triglyceride synthesis, and lipid storage, including *Cidea*, *Cidec*, *Mogat1*, and *CD36* [70]. Restoration of TR4 expression reactivates these pathways, demonstrating a direct role for TR4 in hepatic lipid accumulation [70]. However, under physiological conditions dominated by the PGC-1α coactivator network, TR4 preferentially drives β-oxidation and mitochondrial metabolic programs through recruitment of SRC/p300 complexes and maintenance of an open chromatin state (Figure 2) [36,41]. In this context, TR4 contributes to fatty acid utilization and preservation of hepatic metabolic homeostasis.

Pathological progression appears to occur when the RIP140/HDAC repressive network becomes dominant. RIP140 competitively displaces PGC-1α from TR4 and recruits HDAC3 through its RD1 domain, resulting in deacetylation-dependent repression of β-oxidation and mitochondrial genes [48]. Although TR4 remains transcriptionally active at lipogenic loci such as *SCD1*, *FATP1*, and *CD36* [34,74,75], oxidative metabolic pathways are suppressed, creating a net shift toward lipid uptake and storage. Evidence from hepatocyte-specific RIP140 and HDAC3 models supports a central role for this pathway in hepatic lipid accumulation and progression from simple steatosis to non-alcoholic steatohepatitis (NASH) and fibrosis [56,79]. Moreover, because TR4 also regulates hepatocyte survival through transcriptional control of *Bcl-2*, antagonism of this pathway by RIP140 has been suggested to contribute to inflammation, hepatocellular injury, and fibrogenesis beyond its metabolic effects [44].

Collectively, these findings indicate that the role of TR4 in NAFLD is determined by the balance between PGC-1α-driven oxidative programs and RIP140/HDAC-mediated lipogenic repression (Figure 2). Therapeutic strategies that enhance PGC-1α activity or disrupt the RIP140/HDAC axis may therefore redirect TR4 transcriptional output toward oxidative metabolism and restore its protective functions in hepatic metabolic homeostasis.

### 4.2. Context-Dependent Reprogramming of the TR4 Coregulatory Network in Cancer

TR4 exhibits striking tissue-specific and context-dependent functions across multiple malignancies. In prostate cancer, TR4 functions as a tumor suppressor during tumor initiation but becomes an oncogenic driver during progression to castration-resistant prostate cancer (CRPC) [3,8,80]. In hepatocellular carcinoma (HCC), TR4 generally suppresses metastatic dissemination, although alterations in its associated regulatory network may redirect its activity toward fibrosis and tumor progression [11,12,13]. Similarly, elevated TR4 expression correlates with lymph-node metastasis and poor clinical outcomes in non-small-cell lung cancer (NSCLC), where it promotes EMT and tumor progression [81]. Because genetic alterations of the TR4 locus are relatively uncommon and receptor abundance alone cannot explain these divergent biological outcomes [3,4], increasing evidence suggests that cancer-specific TR4 functions are widely proposed to be primarily determined by dynamic remodeling of its associated coregulatory networks rather than by the receptor itself [8,9].

#### 4.2.1. Prostate Cancer

Prostate cancer provides the clearest example of TR4 functional duality [81,82,83]. During tumor initiation, TR4 acts predominantly as a tumor suppressor by maintaining genomic stability. TR4 directly binds the ATM promoter and activates ATM transcription, thereby supporting DNA damage responses and preventing prostatic intraepithelial neoplasia (PIN) formation [80]. Both TR4-deficient mice and TR4-silenced prostate epithelial cells display reduced ATM expression, increased DNA damage accumulation, and enhanced tumor initiation [80]. At this stage, TR4-mediated tumor suppression appears largely independent of specialized coactivator assemblies such as SRC family members or PGC-1α, relying instead on direct regulation of genome-maintenance pathways [3,80].

As prostate cancer progresses to CRPC, extensive remodeling of the TR4 coregulatory landscape shifts receptor function toward tumor promotion. A central event in this transition is the upregulation of SRC-family coactivators. SRC-3 expression increases during progression from PIN lesions to poorly differentiated carcinomas and promotes prostate cancer cell proliferation and survival, whereas SRC-2/TIF2 expression correlates with AR signaling activity and disease recurrence [63,64,84]. Mechanistically, SRC proteins interact with the activated TR4 LBD through conserved LXXLL motifs and recruit CBP/p300, whose histone acetyltransferase activity enhances chromatin accessibility and transcriptional activation (Figure 2) [37,40,41,62]. TR4 can also be recruited to AR-regulated loci through interaction with AR [14], where it facilitates assembly of SRC-containing activation complexes and amplifies AR-driven transcription through a feed-forward mechanism [8,9]. Consequently, TR4 transcriptional output shifts from maintenance of genomic integrity toward activation of AR signaling, stemness-associated pathways, EMT, and metastatic programs, including EZH2-dependent networks in CD133^+^ prostate cancer stem/progenitor cells [65]. Collectively, these findings indicate that TR4 is converted into a critical driver of CRPC progression.

An additional layer of regulation is provided by JAZF1, a selective TR4-interacting corepressor that occupies the TR4 coactivator-binding interface and competitively limits recruitment of SRC-1 and CBP (Figure 2) [20,68]. However, JAZF1 also exhibits TR4-independent oncogenic functions. Elevated JAZF1 expression promotes EMT, migration, and invasion through activation of the JNK/c-Jun/Slug signaling pathway and can further modulate tumor-associated inflammatory responses through suppression of TAK1-dependent NF-κB signaling [57,85]. Thus, the JAZF1–TR4 axis represents an important regulatory node integrating nuclear receptor signaling, metabolic regulation, and inflammation. Besides protein–protein interactions, TR4 also modulates therapeutic responses via miRNA networks. For instance, TR4 directly binds the promoter of miR-145 to suppress its transcription, thereby de-repressing OCT4 and promoting docetaxel resistance in prostate cancer [58]. This TR4/miR-145/OCT4 axis highlights how TR4, beyond modulating coactivator recruitment, actively regulates chemoresistance through transcriptional repression of tumor-suppressive miRNAs [58]. Collectively, current evidence indicates that the opposing roles of TR4 in prostate cancer are driven not by intrinsic changes in the receptor itself, but by stage-specific remodeling of its surrounding coregulatory network. During tumor initiation, TR4 predominantly supports genomic stability through ATM regulation, whereas during CRPC progression, enhanced SRC-dependent coactivation and associated signaling rewiring redirect TR4 toward oncogenic transcriptional programs [3,4,8,9,80].

#### 4.2.2. Hepatocellular Carcinoma and Metabolism-Driven Malignancies

HCC develops in close association with metabolic dysfunction, chronic inflammation, and extensive transcriptional reprogramming, all of which converge on the TR4 coregulatory network to shape disease-specific transcriptional outputs [86]. Similar to its functions in metabolic disorders and prostate cancer, TR4 activity in HCC is highly context-dependent and determined largely by its associated regulatory partners rather than receptor abundance alone [8,9]. However, unlike the pronounced bidirectional role observed in prostate cancer, TR4 in HCC predominantly exhibits tumor-suppressive and metastasis-restrictive functions while simultaneously influencing chemosensitivity, metabolic adaptation, and fibrogenesis. A central determinant of this functional state is PGC-1α, a master regulator of mitochondrial biogenesis and oxidative metabolism [35]. Because PGC-1α serves as a key coactivator for TR4-dependent oxidative transcriptional programs [36], its frequent downregulation in HCC—which correlates with poor survival, vascular invasion, and larger tumor size—substantially reshapes TR4 transcriptional activity [87,88,89]. Loss of PGC-1α compromises TR4-mediated maintenance of fatty acid oxidation and mitochondrial homeostasis, thereby promoting oxidative stress, lipotoxicity, chronic inflammatory signaling, and a tumor-permissive metabolic environment. Consistent with this model, TR4 suppresses HCC cell migration and invasion through direct repression of *EphA2* [12], enhances cisplatin sensitivity via transcriptional activation of *ATF3* [13], and regulates multiple apolipoprotein genes involved in hepatic lipid homeostasis [90], collectively linking metabolic regulation to tumor suppression.

In parallel, corepressor-associated pathways have been implicated in redirecting TR4 transcriptional outputs toward pathological programs that favor malignant progression. HDAC3, the catalytic component of the NCoR/SMRT repression complex, is essential for maintaining hepatic metabolic and genomic integrity (Figure 2), as liver-specific deletion of HDAC3 ultimately results in spontaneous HCC development [54,55,67]. Excessive HDAC/NCoR-dependent repression may therefore establish an epigenetic environment in which TR4 activity shifts away from programs supporting metabolic homeostasis and differentiation toward those promoting dedifferentiation, proliferation, and tumor progression. Beyond tumor growth, TR4 also contributes to fibrosis by activating the TGF-β1/Smad signaling pathway, increasing p-TβRI and p-Smad2/3 levels while suppressing RXRα expression in hepatic stellate cells [11]. Moreover, TR4 inhibits metastatic dissemination through regulation of the miR-892c-3p/TIMP2 axis [91], a pathway that may be attenuated under conditions of enhanced HDAC/NCoR-mediated silencing. Collectively, current evidence supports a model in which the TR4 coregulatory network has been hypothesized to function as a molecular switch governing phenotypic transitions during hepatocarcinogenesis. Coactivator-dominated states favor oxidative metabolism, cellular differentiation, therapeutic responsiveness, and metastasis suppression, whereas corepressor-dominated states redirect TR4 signaling toward fibrosis, dedifferentiation, proliferation, and malignant progression. This dynamic transition between metabolically differentiated and aggressive tumor states may represent a fundamental mechanism underlying the context-dependent functions of TR4 in HCC.

The contradictory roles of TR4 in cancer largely result from differences in species, disease stages, models, and methods. For instance, TR4 suppresses prostate tumorigenesis via ATM-mediated genomic stability [80], yet promotes CRPC progression through SRC/AR-driven programs [8,9,58,63,64]; similarly, it suppresses HCC invasion in hepatocytes [12,13] but drives fibrosis in stellate cells via TGF-β/Smad2/3 [11]. Discrepancies between in vitro and in vivo outcomes also stem from the loss of immune–stromal crosstalk affecting TR4’s NF-κB pathway [59], while methodological differences in gene manipulation, culture conditions, or detection windows can further lead to inconsistent results. Although similar variability has produced contradictory findings for AR and ERα [92], their ultimate outputs are defined by coactivator/corepressor balances at specific stages rather than by methods themselves. Thus, TR4’s functional duality does not arise from an intrinsic property of the receptor but reflects dynamic remodeling of its coregulatory networks based on tissue context and pathological stage. As summarized in Table 2, while methodological variations are secondary, stage-specific shifts in coactivator/corepressor assembly are the primary determinants. This highlights that targeting the surrounding coregulatory networks, rather than the receptor alone, represents a more rational therapeutic strategy.

### 4.3. Coregulatory Network Remodeling Determines TR4 Functions in Inflammation and Cardiovascular Disease

The role of TR4 in inflammatory disorders is highly context-dependent and cannot be categorized as inherently pro- or anti-inflammatory. Rather, its functional output is heavily influenced by the composition and activity of the surrounding coregulatory network. Depending on the inflammatory milieu, TR4 may either amplify inflammatory signaling and tissue injury or support metabolically protective programs through transcriptional reprogramming. This context dependency is particularly evident in its interaction with NF-κB signaling and in inflammation-associated metabolic diseases (Figure 2), illustrating how dynamic remodeling of TR4-associated coregulator complexes governs disease-specific outcomes.

#### 4.3.1. Crosstalk Between TR4 and NF-κB Signaling Networks

A bidirectional regulatory relationship exists between TR4 and NF-κB, with its functional consequences determined by the associated coregulatory landscape. TNFα suppresses expression of the TR4 target genes *FATP1* and *PC* in 3T3-L1 adipocytes, reducing lipid accumulation through direct interaction of the NF-κB p65 subunit with the TR4 LBD [59]. Unlike JAZF1, which represses TR4 by occupying its coactivator-binding groove, p65 primarily disrupts TR4–DNA interactions, decreasing TR4 occupancy at response elements and attenuating receptor-dependent transactivation [59,61]. Inflammatory signaling further reinforces this effect through induction of HDAC1 and HDAC3, which deacetylate histones at TR4 target loci, promote chromatin condensation, and suppress metabolic gene expression [55,60]. Simultaneously, inflammatory promoters become transcriptionally permissive, resulting in increased expression of cytokines such as IL-6 and TNFα [55]. Consequently, TR4 undergoes a functional shift from regulating metabolic homeostasis to facilitating inflammatory gene expression. Consistent with this model, TR4 directly cooperates with NF-κB p50 to enhance inflammatory promoter occupancy and cytokine production [60]. In sepsis, TR4 expression is elevated in non-survivors, and experimental TR4 overexpression augments IL-1β, IL-6, and TNFα expression, whereas TR4 depletion attenuates inflammatory responses [60]. Similar findings in testicular macrophages support the existence of a feed-forward circuit in which TR4 potentiates NF-κB activity while NF-κB-induced HDAC expression suppresses TR4-dependent metabolic programs, thereby reinforcing inflammatory amplification [60].

JAZF1 serves as a major counter-regulatory node within this network. Through its TID domain, JAZF1 occupies the TR4 coactivator-binding surface and limits TR4-dependent enhancement of NF-κB signaling [61]. In parallel, JAZF1 directly inhibits TAK1-mediated activation of the IKK complex, preventing NF-κB nuclear translocation and suppressing inflammatory cytokine production [57]. Importantly, JAZF1 expression is reduced under high-fat diet conditions [86], removing a critical inhibitory constraint on the TR4–NF-κB positive-feedback loop and facilitating chronic metabolic inflammation. Collectively, these findings suggest that inflammatory functions of TR4 are governed by the balance between HDAC-dependent inflammatory reprogramming and JAZF1-mediated antagonism. Whereas HDAC-associated pathways redirect TR4 activity toward pro-inflammatory transcriptional programs, JAZF1 restrains both TR4 and NF-κB signaling, ultimately determining whether TR4 exerts pathogenic or protective effects in a given inflammatory context (Figure 2).

#### 4.3.2. TR4 Coregulatory Networks in Atherosclerosis

Atherosclerosis is characterized by excessive macrophage lipid uptake, foam cell formation, and chronic vascular inflammation. TR4 contributes directly to these processes by activating *CD36* transcription through binding to TR4REs within the *CD36* promoter, thereby promoting oxidized low-density lipoprotein (ox-LDL) uptake [81]. However, the pathological consequences of TR4 activity are highly dependent on its associated coregulatory network (Figure 2). Under conditions dominated by PGC-1α, macrophages maintain an oxidative and anti-inflammatory phenotype. PGC-1α interacts with activated TR4 through its LXXLL motif and recruits SRC-1 and p300, forming a transcriptional activation complex that promotes histone acetylation and expression of genes involved in fatty acid oxidation and mitochondrial function [41,52,54]. In this setting, TR4-mediated lipid uptake is balanced by efficient oxidative metabolism, preventing excessive lipid accumulation and foam cell formation. PGC-1α also limits TR4–NF-κB interactions, thereby indirectly suppressing inflammatory gene activation [52]. In contrast, dominance of the RIP140/HDAC network fundamentally reprograms macrophage metabolism. Ox-LDL activates TLR4–MAPK signaling, inducing phosphorylation of the TR4 AF-1 domain and promoting a cofactor switch from PCAF to RIP140 [80]. RIP140 subsequently recruits HDAC3 through its RD1 repression domain, leading to histone deacetylation, chromatin compaction, and suppression of oxidative metabolic genes such as *Ucp1* and *Cpt1b* [55,68]. Although TR4 continues to stimulate *CD36* expression under these conditions [82], impaired oxidative capacity results in excessive intracellular lipid accumulation and foam cell formation. Simultaneously, persistent activation of inflammatory genes, including *IL-6* and *TNFα*, further accelerates atherosclerotic progression [55,68].

An additional layer of regulation is provided by ncRNAs (Figure 2). miR-133a directly targets the 3′ untranslated region of TR4 mRNA, reducing TR4 expression and attenuating ox-LDL-induced lipid accumulation and foam cell formation, thereby functioning as an endogenous brake on TR4-driven atherogenic signaling [5]. Collectively, these findings indicate that remodeling of the TR4 coregulatory network—characterized by reduced PGC-1α activity and enhanced RIP140/HDAC signaling—represents a key molecular mechanism underlying the transition from oxidative, anti-inflammatory macrophages to foam-cell-forming, pro-inflammatory macrophages. More broadly, current evidence suggests that TR4 rarely acts as an isolated regulator of disease pathogenesis; instead, its biological functions are defined by dynamic disease-specific coregulatory networks. In metabolic disorders, the balance between PGC-1α and RIP140/HDAC complexes determines whether TR4 promotes energy expenditure or lipid storage [11,52,61,68,93]. In cancer, activation of SRC-family coactivators and loss of inhibitory regulators such as JAZF1 redirect TR4 signaling toward proliferation, invasion, and metastasis [8,80,85]. In inflammatory and cardiovascular diseases, NF-κB-associated pathways together with the RIP140/HDAC axis govern whether TR4 maintains anti-inflammatory homeostasis or amplifies pathological inflammation [57,59,60,74]. Notably, recent studies have expanded the pathological role of TR4 to fibrotic connective tissue diseases. In systemic sclerosis (SSc), TR4 is upregulated via TGF-β/Smad3 signaling and promotes myofibroblast differentiation through a G protein subunit alpha 12 (Gα12)-ROCK-dependent cytoskeletal reorganization [94]. Thus, PGC-1α, SRC family members, RIP140, HDACs, and JAZF1 emerge as central molecular nodes that orchestrate disease-specific reprogramming of TR4 function.

## 5. Epigenetic Governance of TR4 Coregulatory Networks

### 5.1. Chromatin Accessibility as a Primary Determinant of TR4 Genomic Occupancy

**Figure 3:**
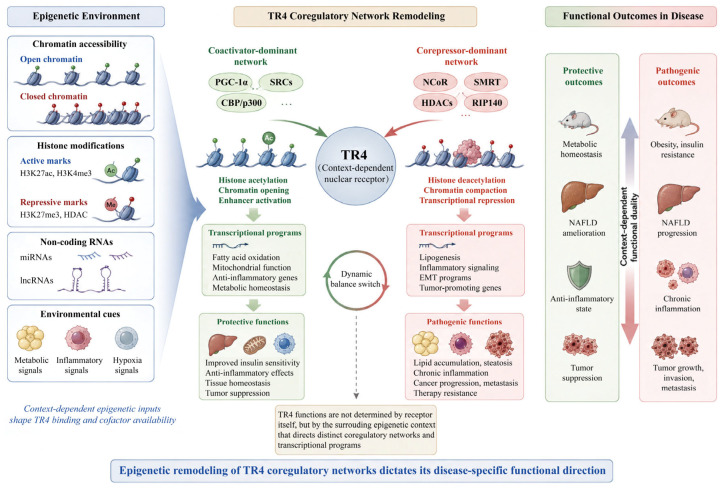
Epigenetic remodeling governs TR4 coregulatory network assembly and functional plasticity. The epigenetic environment acts upstream of TR4 by regulating chromatin accessibility, histone modifications, ncRNA activity, and responsiveness to metabolic or inflammatory cues. These epigenetic inputs shape TR4 genomic binding and influence the recruitment of distinct coactivator or corepressor complexes. Active chromatin states favor assembly of coactivator-dominant networks associated with enhancer activation and metabolic homeostasis, whereas repressive chromatin states facilitate recruitment of corepressor complexes linked to transcriptional silencing and pathogenic gene programs. Through dynamic remodeling of coregulatory network composition, epigenetic mechanisms ultimately determine TR4 transcriptional plasticity and context-dependent functional outcomes.

Although TR4 is capable of recognizing a broad spectrum of TR4REs through its highly conserved DBD, accumulating evidence suggests that DNA sequence alone is insufficient to determine its genomic binding landscape. Similar to many nuclear receptors, TR4-mediated target recognition and transcriptional regulation are highly influenced by the local chromatin environment (Figure 3). Consequently, TR4 does not indiscriminately bind all genomic loci harboring consensus TR4REs; rather, it preferentially occupies regions characterized by accessible chromatin, active enhancers, and ongoing transcriptional activity [30,31,32].

Chromatin accessibility directly influences the ability of TR4 to engage its target loci (Figure 3) [71,72]. Within open chromatin regions, TR4 can readily access DNA and recruit coregulatory complexes to establish transcriptionally competent assemblies. In contrast, highly compacted chromatin domains may remain inaccessible even when canonical TR4-binding motifs are present, thereby preventing effective receptor occupancy. As a result, tissue-specific, developmental-stage-specific, and disease-associated chromatin architectures generate distinct TR4 cistromes and profoundly influence downstream transcriptional programs.

This concept provides a mechanistic explanation for the remarkable tissue specificity and disease specificity of TR4 function. The same TR4 protein exhibits markedly different target-gene preferences in hepatocytes, adipocytes, tumor cells, and inflammatory microenvironments [14,15,16,17,18]. Such differences are not primarily attributable to intrinsic alterations in TR4 itself, but rather arise from cell-type-specific chromatin landscapes that define which regulatory elements are accessible for receptor binding. Therefore, chromatin accessibility not only determines the repertoire of genes available for TR4 regulation but also establishes the molecular framework upon which subsequent coregulatory complexes are assembled. In this regard, chromatin state serves as a critical upstream determinant governing TR4 functional output.

### 5.2. Histone-Modifying Coregulator Networks Shape TR4 Transcriptional Programs

Beyond directing genomic binding-site selection, epigenetic regulation also determines the transcriptional consequences of TR4 occupancy through coordinated histone-modifying networks (Figure 3) [31,32]. Many established TR4 coregulators possess intrinsic chromatin-modifying activities or recruit epigenetic enzyme complexes, thereby linking receptor activity to chromatin remodeling. Coactivators such as SRC family members and CBP/p300 promote histone acetylation, chromatin accessibility, and enhancer activation, thereby facilitating TR4-dependent programs involved in fatty acid oxidation, mitochondrial homeostasis, cellular differentiation, and anti-inflammatory responses [19,20,30,31,32]. In contrast, corepressor complexes including NCoR, SMRT, and HDACs induce histone deacetylation and chromatin compaction, suppressing metabolic homeostatic programs while favoring lipid accumulation, chronic inflammation, cellular dedifferentiation, and tumor progression [21,22]. Accordingly, TR4 has been proposed to function not only as a sequence-specific transcription factor but also as an epigenetic scaffold that integrates extracellular signals, metabolic cues, and intracellular stress responses through selective recruitment of chromatin-modifying complexes. The biological outcome of TR4 signaling therefore depends less on the receptor itself than on the epigenetic machinery assembled around it. Differential recruitment of histone-modifying complexes provides a mechanistic basis for disease-specific TR4 transcriptional programs and contributes substantially to the context-dependent nature of TR4 function.

### 5.3. NcRNAs as Dynamic Modulators of TR4 Coregulatory Networks

ncRNAs have emerged as important intermediates linking epigenetic regulation with transcription-factor activity [95,96,97]. Accumulating evidence suggests that microRNAs (miRNAs), long non-coding RNAs (lncRNAs), and other regulatory RNA species are not merely downstream targets of TR4 signaling but active participants in shaping TR4 coregulatory networks (Figure 3). miRNAs can directly regulate TR4 expression or indirectly modulate its transcriptional activity by targeting key coregulatory components, including PGC-1α, HDACs, NCoR, and related regulatory nodes, thereby influencing the balance between coactivator and corepressor networks and altering the directionality of TR4-dependent transcriptional programs [98,99,100]. In parallel, lncRNAs function as molecular scaffolds, decoys, guides, or chromatin organizers that facilitate or disrupt interactions between TR4 and specific coregulators, affecting chromatin accessibility, enhancer activity, transcriptional complex assembly [101], and, in some cases, higher-order chromatin organization and enhancer–promoter communication [102]. Thus, ncRNAs should be regarded not simply as downstream effectors of TR4 signaling but as dynamic network modifiers that integrate metabolic status, inflammatory signals, and environmental stimuli into the TR4 regulatory framework, providing an additional layer of plasticity that contributes to the context dependency and functional adaptability of TR4 signaling.

### 5.4. Epigenetic Context as the Molecular Basis of TR4 Functional Duality

A longstanding question in the TR4 field is why the same nuclear receptor can exert seemingly opposing biological effects across different diseases—or even at different stages of the same disease. Emerging evidence suggests that this functional duality does not arise from intrinsic alterations in TR4 itself but from differences in the surrounding epigenetic environment (Figure 3). Although the structural integrity of TR4 remains largely unchanged across pathological contexts, tissue- and disease-specific patterns of chromatin accessibility, histone modifications, ncRNA expression, and coregulator abundance collectively dictate the transcriptional complexes that TR4 can assemble, thereby shaping target-gene selection and transcriptional output (Figure 3) [19,20,21,22]. Thus, the apparent functional duality of TR4 reflects the dynamic assembly of distinct coregulatory networks under different epigenetic conditions rather than an intrinsic contradiction within the receptor itself. This framework provides a unifying explanation for the diverse roles of TR4 in metabolic disorders, cancer, and chronic inflammatory diseases, while highlighting epigenetic remodeling as a central determinant of TR4 function. It also points to a conceptual shift in therapeutic strategy—from directly targeting TR4 to modulating the epigenetic landscape and coregulatory networks that dictate its activity, thereby influencing disease initiation, progression, and therapeutic responsiveness.

## 6. Therapeutic Reprogramming of TR4 Signaling: From Receptor-Centric Targeting to Coregulatory Network Intervention

The expanding recognition of TR4, as a critical regulator of metabolic homeostasis, tumor progression, and chronic inflammatory disorders, has generated considerable interest in its therapeutic potential. However, unlike classical ligand-regulated nuclear receptors, TR4 exhibits remarkable functional heterogeneity across tissues and pathological contexts and may even exert opposing biological effects during different stages of the same disease [11,12,13]. This context-dependent behavior challenges the traditional receptor-centric paradigm that has successfully guided the development of therapeutics targeting ER, AR, peroxisome proliferator-activated receptors (PPARs), and farnesoid X receptor (FXR). As summarized throughout this review, disease-specific TR4 activity is determined not only by the receptor itself but, more importantly, by the composition and functional state of the surrounding coregulatory network. Consequently, a conceptual shift is emerging—from directly targeting TR4 to selectively modulating the molecular networks that govern its transcriptional output. This network-oriented perspective may provide a more effective and biologically rational framework for therapeutic intervention in TR4-associated diseases.

### 6.1. Challenges and Limitations of Direct TR4 Targeting

Nuclear receptors have long represented one of the most successful classes of druggable transcription factors, with numerous approved therapeutics targeting ER, AR, PPARs, and FXR [103,104,105]. In contrast, progress toward pharmacological targeting of TR4 has remained limited. A major challenge is its classification as an orphan nuclear receptor, as no universally accepted endogenous ligand has yet been identified. Although several lipid metabolites, unsaturated fatty acids, and metabolically derived compounds have been reported to influence TR4 activity, their direct binding mechanisms, receptor specificity, and physiological relevance remain incompletely defined [2], limiting the applicability of conventional ligand-screening strategies. Notably, recent drug repurposing screening has identified direct small-molecule regulators of TR4, with nilotinib as a potent inhibitor and genistein as an activator [106]. However, these repurposed compounds lack TR4 selectivity and exhibit broad off-target effects as kinase inhibitors or phytoestrogens [106]. Thus, achieving receptor-specific targeting without unintended crosstalk remains a major obstacle for clinical translation. More importantly, TR4 displays profound context-dependent functional plasticity. In metabolic tissues, TR4 may support mitochondrial function and fatty acid oxidation under some conditions while promoting lipid accumulation, insulin resistance, and chronic inflammation under others [61,68,73,74,75,76,77]. Similarly, in cancers such as prostate cancer, the consequences of TR4 activation vary substantially according to disease stage and the surrounding transcriptional regulatory landscape [8,9,64]. Consequently, indiscriminate activation or inhibition of TR4 carries the risk of simultaneously affecting both beneficial and pathogenic transcriptional programs, creating substantial uncertainty regarding therapeutic outcomes (Table 1, Figure 2). These observations suggest that TR4 should be regarded less as a conventional therapeutic target with fixed biological functions and more as a conditional transcriptional regulator whose activity is continuously shaped by its molecular environment. Preserving beneficial TR4 functions while selectively suppressing disease-promoting programs therefore remains a central challenge for TR4-targeted therapy.

### 6.2. Coregulatory Networks as Therapeutic Targets: A New Intervention Paradigm

Evidence discussed in Chapters 3 and 4 consistently demonstrates that TR4 function is ultimately determined by the coregulatory complexes assembled at target chromatin regions rather than by receptor abundance alone. Depending on tissue type and disease context, TR4 engages distinct combinations of metabolic coactivators, inflammatory corepressors, and environmentally responsive regulators, generating transcriptional programs that are often not merely different but functionally antagonistic [18,19,20,21,22]. This insight fundamentally reframes the therapeutic question: rather than asking whether TR4 activity should be increased or decreased, a more relevant objective is how to redirect TR4 toward beneficial transcriptional outputs by manipulating critical nodes within its coregulatory network. Among currently identified regulatory modules, epigenetic corepressor complexes are particularly attractive therapeutic targets. NCoR-, SMRT-, and HDAC-containing complexes establish repressive chromatin environments that suppress genes involved in metabolic homeostasis while facilitating lipid accumulation, chronic inflammation, and tumor progression (Table 1, Figure 3) [51,52]. Given the extensive evidence linking aberrant HDAC activity to obesity, NAFLD, and multiple malignancies [48,49,50], clinically approved HDAC inhibitors such as Vorinostat, Panobinostat, and Romidepsin may partially exert therapeutic effects through remodeling TR4-associated transcriptional networks [107,108,109], despite not having been developed specifically for TR4. Additionally, small-molecule inhibitors targeting other key nodes of the TR4 coregulatory network, such as CBP/p300 (e.g., C646) and NF-κB (e.g., BAY 11-7082), have been reported to exert anticancer activities [110,111]. These compounds hold potential to reprogram TR4-dependent transcription in cancer and inflammatory diseases, although their clinical application remains largely restricted to preclinical or early-stage studies.

Another important therapeutic module is the PGC-1α-centered metabolic coactivator network. As a master regulator of mitochondrial biogenesis and oxidative metabolism, PGC-1α promotes TR4-dependent transcriptional programs involved in fatty acid oxidation, mitochondrial function, and energy expenditure [35,36]. Although no approved therapies directly target PGC-1α, agents such as metformin, resveratrol, AMPK activators, and SIRT1-modulating strategies can indirectly enhance its activity [112,113,114]. Similarly, targeting the SRC family of coactivators has been explored using small-molecule inhibitors (e.g., targeting SRC-3 or upstream kinases), which may limit TR4-mediated tumor progression in contexts like prostate cancer [63,64]. Importantly, the goal of such interventions is not to globally activate TR4 but to bias its transcriptional output toward oxidative and metabolically beneficial programs. Environment-responsive regulatory networks also represent emerging opportunities. In particular, JAZF1 has attracted increasing attention following genome-wide association studies linking JAZF1 polymorphisms to T2D, obesity, and metabolic syndrome susceptibility [115]. As a key TR4-interacting protein, JAZF1 influences receptor activity, complex stability, and target-gene selection, thereby serving as a molecular bridge between genetic predisposition, metabolic regulation, and nuclear receptor signaling. Although direct pharmacological modulation of JAZF1 is not yet available, the broader strategy of targeting critical network nodes rather than the receptor itself represents an important conceptual advance.

Despite the conceptual appeal of network-based strategies, several challenges must be addressed. The major advantage is the ability to selectively reprogram TR4 outputs rather than simply activating or inhibiting global TR4 activity. However, the primary drawbacks lie in off-target effects and tissue specificity. Since key coregulators such as HDAC3, p300, PGC-1α, and NF-κB participate in multiple cellular pathways and are broadly expressed across tissues, systemic inhibition carries a significant risk of unintended toxicities. Therefore, achieving tissue- and disease-specific targeting of these networks (e.g., through drug delivery systems or local administration) is critical for clinical translation. At present, no selective, direct small-molecule modulators of TR4 are available for clinical use. While recent drug repurposing efforts have identified nilotinib and genistein as TR4 regulators, their lack of selectivity for TR4 over other kinases and nuclear receptors limits their therapeutic utility [113]. Consequently, developing highly specific direct TR4 regulators, or contextually restricted network inhibitors, remains a major frontier for TR4-targeted precision therapy.

### 6.3. Precision Medicine Through TR4 Coregulatory Landscape Profiling

A major implication emerging from Chapter 4 is that TR4 function cannot be reliably predicted by receptor expression levels alone. Even within the same disease, distinct coregulatory environments may drive TR4 toward opposite biological outcomes. In metabolic tissues, TR4 associated with a PGC-1α-dominant coactivator network preferentially promotes fatty acid oxidation, mitochondrial activity, and metabolic homeostasis [19,20], whereas coupling with RIP140–HDAC corepressor complexes redirects TR4 toward transcriptional programs that favor lipid accumulation, metabolic dysfunction, and chronic inflammation [21,22]. Similar context-dependent switching has been observed in multiple cancers, where different combinations of coactivators and corepressors determine whether TR4 exerts tumor-suppressive or tumor-promoting functions [8,9]. These observations suggest that the TR4 coregulatory landscape may provide a more accurate indicator of receptor function than TR4 expression alone.

From a clinical perspective, patients with comparable levels of TR4 expression or activation may nevertheless possess fundamentally different disease-driving mechanisms. In one patient, TR4 activity may primarily reflect engagement with oxidative metabolic coactivators such as PGC-1α and support tissue homeostasis; in another, TR4 may predominantly associate with RIP140, HDACs, or inflammatory signaling networks, thereby promoting pathogenic transcriptional outputs. Under such circumstances, identical TR4-directed interventions could yield markedly different—or even opposing—clinical outcomes. A more rational precision-medicine framework would therefore focus on defining TR4 coregulatory signatures that integrate coactivator abundance, corepressor composition, chromatin accessibility, inflammatory signaling activity, and epigenetic states within diseased tissues. Such signatures could identify the regulatory environment governing TR4 activity and facilitate individualized therapeutic selection.

Recent advances in transcriptomics, proteomics, single-cell sequencing, spatial transcriptomics, and systems biology are making this approach increasingly feasible. These technologies enable disease classification based on regulatory-network states rather than individual gene expression levels, providing a more functionally relevant view of disease biology. Nevertheless, important challenges remain. Most TR4-associated coregulators participate in multiple transcriptional systems and are broadly expressed across tissues [65,92,116], raising concerns regarding specificity and off-target effects. In addition, many TR4 regulatory axes and dynamic protein-interaction networks remain incompletely characterized, and the mechanisms governing network remodeling across disease stages, tissue compartments, and cellular subpopulations are still poorly understood. Addressing these questions will require integrated application of single-cell multi-omics, spatial molecular profiling, epigenomic mapping, and dynamic interactome analyses to define the spatiotemporal architecture of TR4 coregulatory networks.

At a broader conceptual level, advances in TR4 biology support a paradigm shift in nuclear receptor therapeutics. The key determinant of disease phenotype and treatment response may not be the receptor itself, but the regulatory network in which it operates. Accordingly, the future of TR4-targeted therapy may lie less in the search for novel agonists or antagonists and more in the identification and selective reprogramming of the molecular networks that dictate TR4 function. At the interface of experimental biology and clinical translation, advanced mathematical modeling of tumor–drug interactions has also provided valuable predictive frameworks for optimizing multi-agent combination therapies [117]. As systems biology and multi-omics technologies continue to evolve, network-guided precision intervention is poised to become a promising strategy for metabolic disorders, cancer, and chronic inflammatory diseases, while also providing a broader framework for understanding and therapeutically manipulating nuclear receptor signaling in complex human diseases.

## 7. Conclusions and Perspectives

TR4 is an orphan nuclear receptor that participates extensively in the regulation of metabolic homeostasis, inflammatory responses, cellular differentiation, and tumorigenesis. Despite intensive investigation over the past two decades, one of the most intriguing features of TR4 biology remains its remarkable functional plasticity. Across metabolic diseases, cancer, and chronic inflammatory disorders, TR4 can exert either protective or pathogenic effects depending on the cellular and pathological context. The evidence summarized in this review suggests that this apparent functional duality is not an intrinsic property of the receptor itself but rather reflects the dynamic regulatory environment in which TR4 operates. Coactivators, corepressors, chromatin-remodeling complexes, epigenetic modifiers, and environmentally responsive signaling pathways collectively form an integrated coregulatory network that governs TR4 activity at multiple levels. Through coordinated regulation of receptor conformation, chromatin accessibility, enhancer activity, and target-gene selection, these factors ultimately determine the transcriptional output and biological consequences of TR4 signaling. Accordingly, TR4 should be viewed not simply as a nuclear receptor with predetermined functions but as a transcriptional regulatory platform whose activity is continuously shaped by its surrounding molecular network.

This perspective supports a conceptual shift from a traditional receptor-centered view toward a network-centered model of TR4 biology. Rather than being dictated by receptor abundance alone, disease-specific TR4 functions are defined by the composition and functional state of the coregulatory complexes assembled around the receptor. Such a framework provides a unified explanation for the long-standing paradox of TR4 functional duality and further suggests that common regulatory principles may underlie the involvement of TR4 in seemingly distinct pathological conditions, including metabolic dysfunction, chronic inflammation, fibrosis, and cancer. More importantly, it implies that the critical determinants of disease progression are not the receptor itself but the regulatory networks that direct its transcriptional programs.

Looking ahead, future studies will likely move beyond the characterization of individual TR4-interacting factors toward the systematic mapping of disease-specific TR4 coregulatory landscapes. Achieving this goal will require the integration of emerging genomic, epigenomic, and spatial profiling technologies. Chromatin profiling approaches such as ChIP-seq, CUT&RUN/CUT&Tag, and ATAC-seq will facilitate high-resolution characterization of TR4 genomic occupancy, chromatin accessibility, and dynamic coregulator recruitment across different disease contexts. Meanwhile, single-cell RNA sequencing, single-cell multi-omics, and spatial transcriptomics will enable the dissection of TR4 regulatory networks with unprecedented cellular and spatial resolution, allowing for the identification of cell type-specific transcriptional programs and microenvironment-dependent regulatory interactions. Combined with dynamic interactome analyses, these approaches are expected to define the spatiotemporal architecture of TR4 coregulatory networks during disease initiation and progression and to identify critical regulatory hubs linking genetic susceptibility, metabolic reprogramming, inflammatory signaling, and tumor evolution. From a translational perspective, the framework proposed here also argues for a new therapeutic paradigm in which intervention strategies focus on selectively remodeling TR4-associated coregulatory networks rather than simply activating or inhibiting the receptor itself. By targeting key regulatory nodes and epigenetic mechanisms that determine TR4 transcriptional output, it may become possible to redirect disease-associated programs toward beneficial outcomes with greater precision and fewer adverse effects. Collectively, these advances have the potential not only to redefine our understanding of TR4 biology but also to establish a broader conceptual framework for network-guided therapeutic modulation of nuclear receptor signaling in complex human diseases.

## Figures and Tables

**Table 2:** Dual roles of TR4 in human cancers and underlying coregulatory mechanisms.

Cancer Type	Role of TR4	Disease Stage/Cell Context	Coregulatory Network/Mechanism
Prostate Cancer	Suppression	Early stage: prostatic intraepithelial neoplasia (PIN)	ATM-mediated DNA damage repair (genomic stability) ^a^ [80]
	Promotion	Late stage: castration-resistant PCa (CRPC)	SRC family coactivators / AR signaling axis ^a^ [8,9,64,65]; EZH2-mediated stemness ^a^ [65] and miR-145/OCT4 axis (chemoresistance) ^a^ [58]
Hepatocellular Carcinoma (HCC)	Suppression	Malignant hepatocytes (primary & metastatic)	PGC-1α oxidative metabolism axis ^a^ [12,13]; ATF3-mediated chemosensitization ^a^ [13]; inhibition of EphA2 invasion ^a^ [12]
	Pro-fibrotic/Tumor promotion	Non-tumor microenvironment (Hepatic stellate cells)	TGF-β receptor I/Smad2/3 signaling pathway ^a^ [11]; NCoR/HDAC3 epigenetic repression ^b^ [67]
Non-Small-Cell Lung Cancer (NSCLC)	Promotion	Advanced disease with lymph node metastasis	EMT (epithelial–mesenchymal transition) activation (pro-metastatic reprogramming) ^a^ [81]
Renal Cell Carcinoma (RCC)	Promotion	Metastatic clear cell RCC	HGF/MET signaling ^a^ [24] (under post-transcriptional regulation by miR-32-5p); vasculogenic mimicry ^a^ [24]

Evidence levels are defined as follows: ^a^ established experimental evidence; ^b^ evidence inferred from related nuclear receptors.

## Data Availability

No new data were created or analyzed in this study. Data sharing is not applicable to this article.
